# Interferon-γ enhances the therapeutic effect of mesenchymal stem cells on experimental renal fibrosis

**DOI:** 10.1038/s41598-020-79664-6

**Published:** 2021-01-13

**Authors:** Ryo Kanai, Ayumu Nakashima, Shigehiro Doi, Tomoe Kimura, Ken Yoshida, Satoshi Maeda, Naoki Ishiuchi, Yumi Yamada, Takeshi Ike, Toshiki Doi, Yukio Kato, Takao Masaki

**Affiliations:** 1 Department of Nephrology, Hiroshima University Hospital, 1-2-3 Kasumi, Minami-ku, Hiroshima, Hiroshima 734-8551 Japan; 2 Department of Stem Cell Biology and Medicine, Graduate School of Biomedical and Health Sciences, Hiroshima University, 1-2-3 Kasumi, Minami-ku, Hiroshima, Hiroshima 734-8553 Japan; 3 TWOCELLS Company, Limited, 16-35 Hijiyama-honmachi, Minami-ku, Hiroshima, 732-0816 Japan

**Keywords:** Cell biology, Stem cells, Nephrology

## Abstract

Mesenchymal stem cells (MSCs) administered for therapeutic purposes can be activated by interferon-γ (IFN-γ) secreted from natural killer cells in injured tissues and exert anti-inflammatory effects. These processes require a substantial period of time, leading to a delayed onset of MSCs’ therapeutic effects. In this study, we investigated whether pretreatment with IFN-γ could potentiate the anti-fibrotic ability of MSCs in rats with ischemia–reperfusion injury (IRI) and unilateral ureter obstruction. Administration of MSCs treated with IFN-γ strongly reduced infiltration of inflammatory cells and ameliorated interstitial fibrosis compared with control MSCs without IFN-γ treatment. In addition, conditioned medium obtained from IFN-γ-treated MSCs decreased fibrotic changes in cultured cells induced by transforming growth factor-β1 more efficiently than that from control MSCs. Most notably, secretion of prostaglandin E2 from MSCs was significantly increased by treatment with IFN-γ. Increased prostaglandin E2 in conditioned medium obtained from IFN-γ-treated MSCs induced polarization of immunosuppressive CD163 and CD206-positive macrophages. In addition, knockdown of prostaglandin E synthase weakened the anti-fibrotic effects of MSCs treated with IFN-γ in IRI rats, suggesting the involvement of prostaglandin E2 in the beneficial effects of IFN-γ. Administration of MSCs treated with IFN-γ might represent a promising therapy to prevent the progression of renal fibrosis.

## Introduction

The morbidity rate of chronic kidney disease (CKD) is estimated to be 8%–16% worldwide^1^. Etiological studies of CKD have reported multiple causes of disease initiation including hypertension, diabetes mellitus, and glomerulonephritis^2^. Despite differences in disease initiation, renal fibrosis exacerbated by persistent inflammation is a histological change common to all these etiologies^3,4^. Currently there are few effective treatments that prevent the progression of CKD and many patients eventually develop renal failure, which requires renal replacement therapy, resulting in a heavy social and economic burden. Therefore, there is an urgent need for the development of novel therapeutic strategies to treat renal fibrosis associated with CKD.

The pathogenesis of CKD is mediated by inflammatory cells, which cause renal fibrosis via fibroblast activation and increased extracellular matrix deposition^5^. Damage-associated molecular patterns (DAMPs) released from damaged tissues activate the local immune system and several studies reported DAMPs were involved in promoting renal fibrosis^6–8^. In CKD patients, the inflammatory microenvironment is maintained by infection, uremic toxins, or tissue ischemia^9^, and this chronic inflammation contributes to the sustained release of DAMPs from injured kidney tissues, which induces further inflammation and fibrosis. Therefore, the inhibition of inflammation is expected to ameliorate renal fibrosis.

Mesenchymal stem cells (MSCs) isolated from various tissues including bone marrow, blood, and adipose tissue^10^, have multipotency and self-renewal ability^11,12^. They exert their beneficial effects by suppressing inflammation and fibrosis via a paracrine mechanism^13,14^. Several studies have reported that MSCs or extracellular vesicles derived from MSCs have beneficial effects for renal fibrosis^15–18^.

High mobility group box-1 protein (HMGB1) and interleukin-18 (IL-18) are members of DAMPs— HMGB1 was reported to promote the migration of MSCs^19,20^, whereas IL-18 contributed to the secretion of interferon-γ (IFN-γ) from natural killer cells^21^. Furthermore, IFN-γ released from immune cells at sites of damaged tissues stimulated MSCs to secrete anti-inflammatory mediators including prostaglandin E2 (PGE2)^22–26^. PGE2 has been reported to induce the polarization of immunosuppressive M2 macrophages that produce anti-inflammatory cytokines and inhibit the persistence of inflammation^27,28^. In addition, we previously reported that MSCs promoted macrophage differentiation from an M1 pro-inflammatory phenotype to an immunosuppressive M2 phenotype, and that the administration of ex vivo-expanded MSCs suppressed the progression of fibrosis^29,30^. Taken together, this suggests that IFN-γ-preconditioned MSCs have a strong immunosuppressive effect and therefore might ameliorate renal fibrosis. However, the utility of MSCs cultured with IFN-γ for the treatment of kidney disease has not been investigated.

This study investigated the therapeutic effect of MSCs cultured in IFN-γ-containing medium on inflammation and fibrosis using rat ischemia–reperfusion injury (IRI) and unilateral ureter obstruction (UUO) models.

## Results

### Expression of HMGB1, IL-18, and IFN-γ in the kidney after the IRI procedure

**Figure 1:**
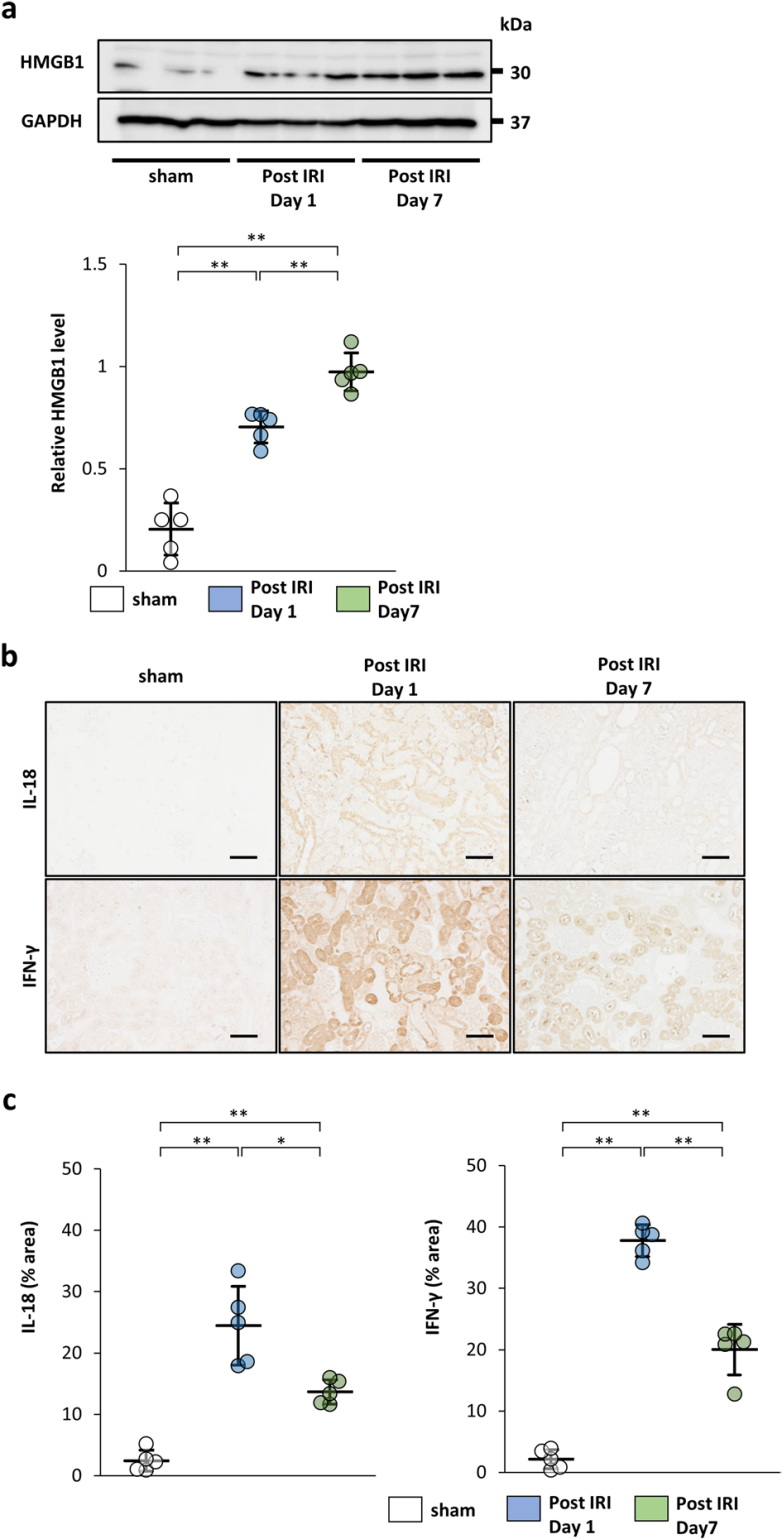
Expression of high mobility group box-1 protein (HMGB1), interleukin-18 (IL-18), and interferon (IFN)-γ in the kidney after the ischemia–reperfusion injury (IRI) procedure. Rats were sacrificed at 1 day (Post IRI Day 1) and 7 days (Post IRI Day 7) after the IRI procedure and evaluated for expression of HMGB1, IL-18, and IFN-γ. (**a**) Western blot analysis of HMGB1 in the rat kidney cortex. Protein levels were normalized to GAPDH levels (n = 5 in each group). The blots are the cropped images from different parts of the same gel. Full-length gel images are provided in the supplementary file. The samples derive from the same experiment and that gels were processed in parallel. (**b**) Representative immunohistochemical staining of IL-18 and IFN-γ in kidney sections (scale bar, 100 µm). (**c**) Quantification of IL-18 and IFN-γ-positive areas (n = 5 in each group). Data are presented as the mean ± SD. *p < 0.05, **p < 0.01. Sham, non-IRI procedure.

HMGB1 and IL-18, which are known as DAMPs, are released from damaged tissues. Released IL-18, which is also induced by HMGB1^8^, contributes to secretion of IFN-γ^19^. To confirm expression of HMGB1, IL-18, and IFN-γ induced by IRI, rats were sacrificed to evaluate their expression in the kidney at 1 day (Post IRI Day 1) and 7 days (Post IRI Day 7) after the IRI procedure. As shown in a previous study^31^, the protein level of HMGB1 was increased by the IRI procedure (Fig. 1a). Immunostaining revealed that IL-18 and IFN-γ-positive areas were increased strongly in the kidney at 1 day after IRI. However, their increase was attenuated at 7 days after IRI (Fig. 1b,c). These changes are similar to those seen in a previous study^32^. Furthermore, because IL-18 promotes IFN-γ secretion from natural killer cells, IFN-γ expression might be associated with IL-18 secretion. MSCs are activated by released IFN-γ and exert immunosuppressive effects^22–26^. Therefore, administration of MSCs cultured in IFN-γ-containing medium might have beneficial effects on preventing the progression of renal fibrosis.

### IFN-γ enhances the ability of MSCs to attenuate fibrosis induced by IRI

**Figure 2:**
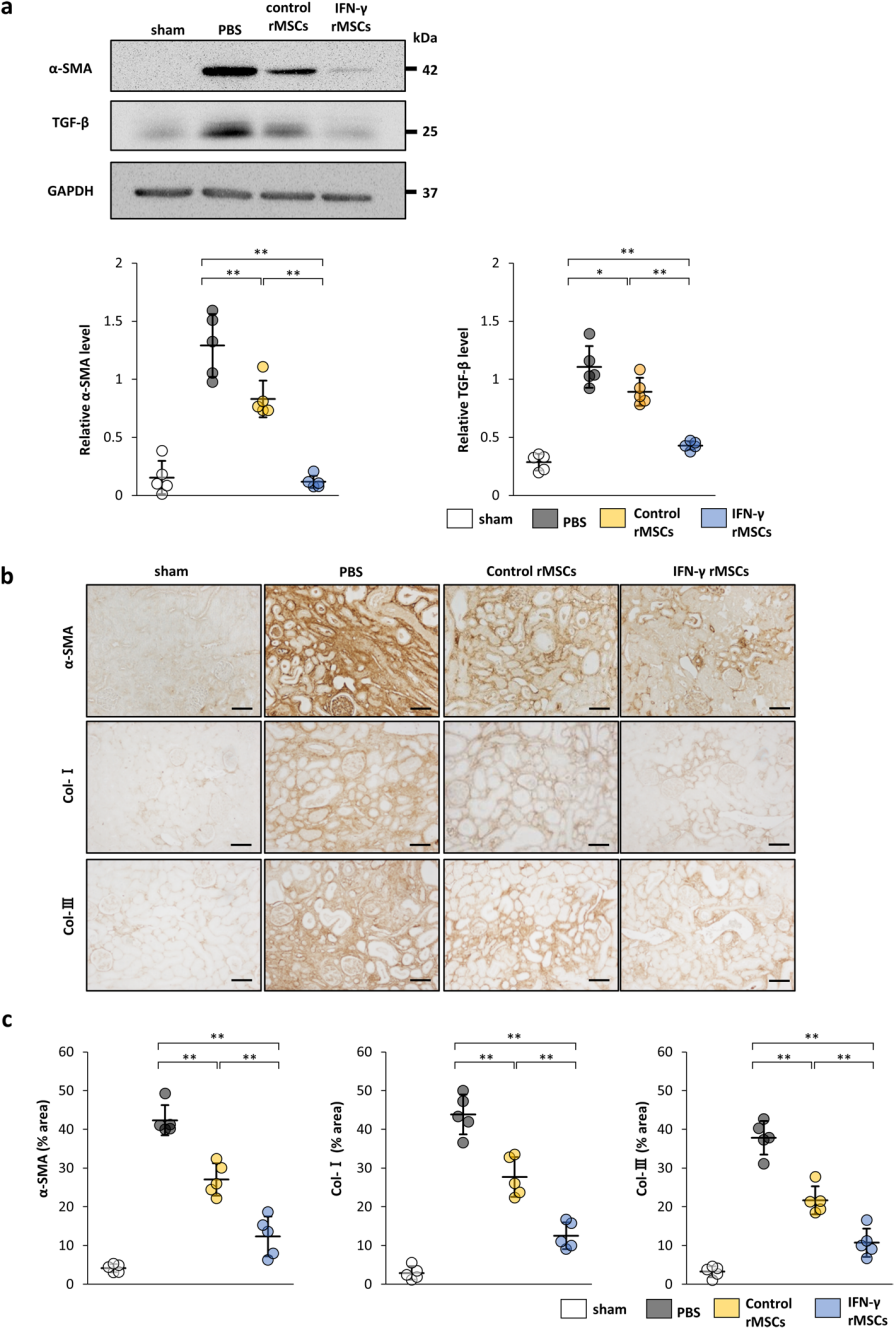
Anti-fibrotic effects of IFN-γ-treated mesenchymal stem cells (MSCs) in the kidney of IRI rats. IFN-γ-treated or untreated rat MSCs were injected immediately after IRI induction. Twenty-one days later, protein levels of fibrosis markers were evaluated by western blot (**a**) and immunohistochemical (**b**, **c**) analyses. (**a**) Western blot analysis of α-SMA and TGF-β1 in the rat kidney cortex. Protein levels were normalized to GAPDH levels (n = 5 in each group). The blots are the cropped images from different parts of the same gel. Full-length gel images are provided in the supplementary file. The samples derive from the same experiment and that gels were processed in parallel. (**b**) Representative immunohistochemical staining of α-SMA, Collagen type I (Col-I), and Collagen type III (Col-III) in kidney sections. (scale bar, 100 µm). (**c**) Quantification of α-SMA-, Col-I-, and Col-III-positive areas (n = 5 in each group). Data are presented as the mean ± SD. **p < 0.01. Sham, non-IRI procedure; PBS, PBS injection; control rMSCs, rat MSCs injection; IFN-γ rMSCs, injection of IFN-γ-treated rMSCs.

To evaluate anti-fibrotic effects of MSCs, we injected PBS, rat MSCs (rMSCs) treated with IFN-γ (IFN-γ rMSCs) or untreated rMSCs (control rMSCs) into the abdominal aorta of rats after ischemic reperfusion. Twenty-one days later, the rats were sacrificed, and injured kidneys were collected to evaluate the degree of fibrosis by western blotting. The protein levels of α-smooth muscle actin (α-SMA) and transforming growth factor-β1 (TGF-β1), markers for drivers of fibrosis, were increased in the kidney of PBS-injected rats and their levels were suppressed by injection of control rMSCs (Fig. 2a). Furthermore, injection of IFN-γ rMSCs decreased IRI-induced fibrotic changes more significantly than that of control rMSCs (Fig. 2a). Immunostaining of α-SMA, collagen type I (Col-I), and collagen type III (Col-III) (extracellular matrix proteins) was also performed to assess renal fibrosis. α-SMA, Col-I, and Col-III-positive areas were increased in the PBS group. Similar to the results from western blotting, administration of IFN-γ rMSCs reduced α-SMA, Col-I, and Col-III-positive areas more strongly compared with that of control rMSCs (Fig. 2b,c). These results suggest that IFN-γ-preconditioned rMSCs have a strong anti-fibrotic effect.

### IFN-γ enhances the ability of MSCs to inhibit inflammation induced by IRI

**Figure 3:**
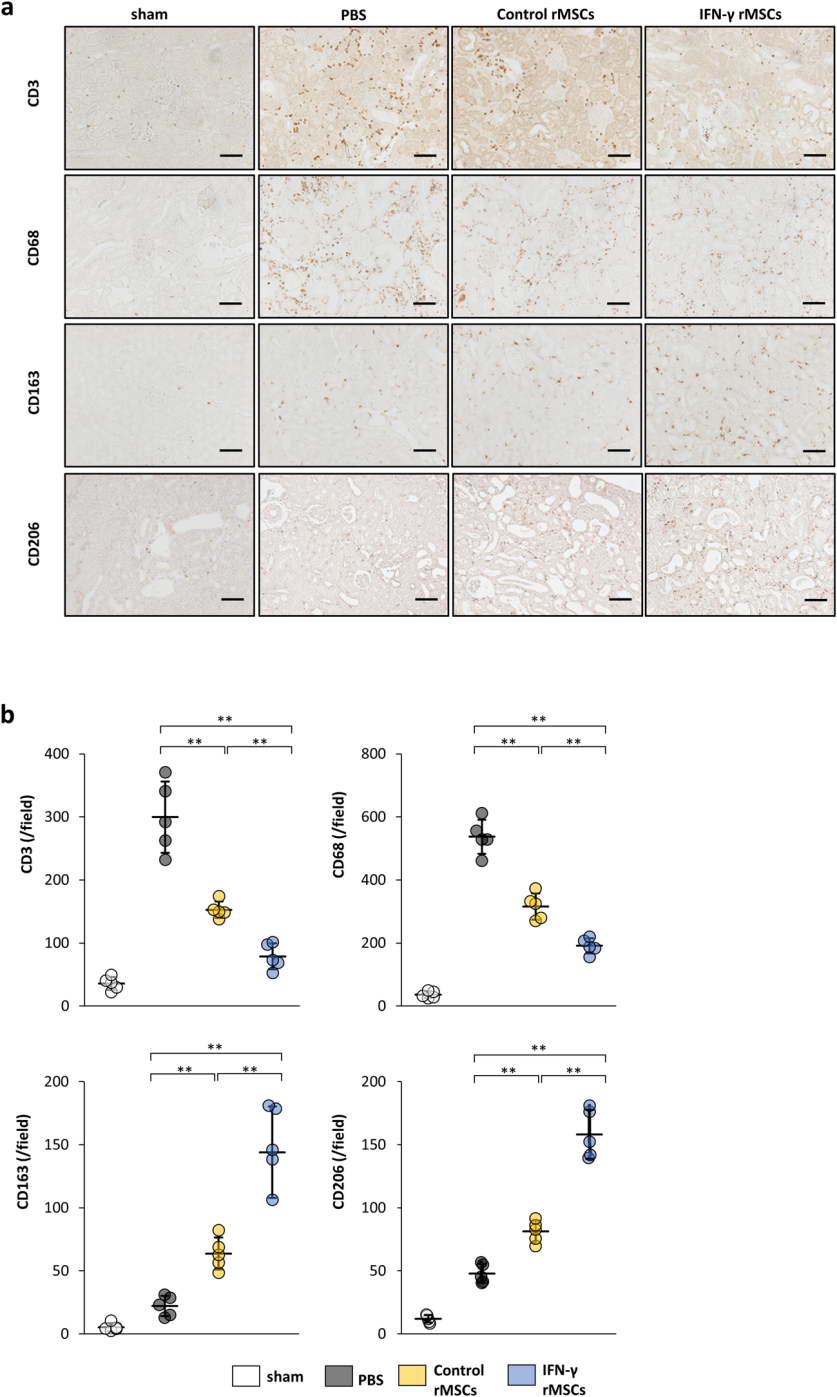
Anti-inflammatory effects of IFN-γ-treated MSCs in IRI rats. Infiltration of T cells and macrophages into injured kidney of MSC-injected IRI rats was assessed at 7 days after IRI by immunostaining. (**a**) Representative immunohistochemical staining of CD3, CD68, CD163 and CD206 in rat kidney sections (scale bar, 100 µm). (**b**) Quantification of CD3-, CD68-, CD163- and CD206-positive cells (n = 5 in each group). Data are presented as the mean ± SD. **p < 0.01. Abbreviations are as in Fig. 2.

As shown above, IFN-γ rMSCs strongly suppressed renal fibrosis in IRI rats. We next examined the anti-inflammatory effects of IFN-γ rMSCs in the acute phase of renal injury in IRI rats. rMSCs with or without IFN-γ treatment were injected into rats after IRI induction, and 7 days later, the rats were sacrificed to investigate infiltration of inflammation cells into the kidney. Immunostaining showed that infiltration of CD3 (T cell marker)- and CD68 (macrophage marker)-positive cells was markedly increased in the PBS group (Fig. 3a,b). Injection of control rMSCs suppressed the infiltration of these cells, and the suppression was more significant in the IFN-γ rMSCs group (Fig. 3a,b). However, the infiltration of CD163 and CD206 (immunosuppressive macrophage markers)-positive cells was increased by injection of control rMSCs, and injection of IFN-γ rMSCs resulted in a more significant increase in CD163 and CD206-positive cells (Fig. 3a,b), indicating that IFN-γ-preconditioned rMSCs have a stronger anti-inflammatory effect.

### Administration of IFN-γ-preconditioned MSCs strongly attenuates UUO-induced fibrosis

**Figure 4:**
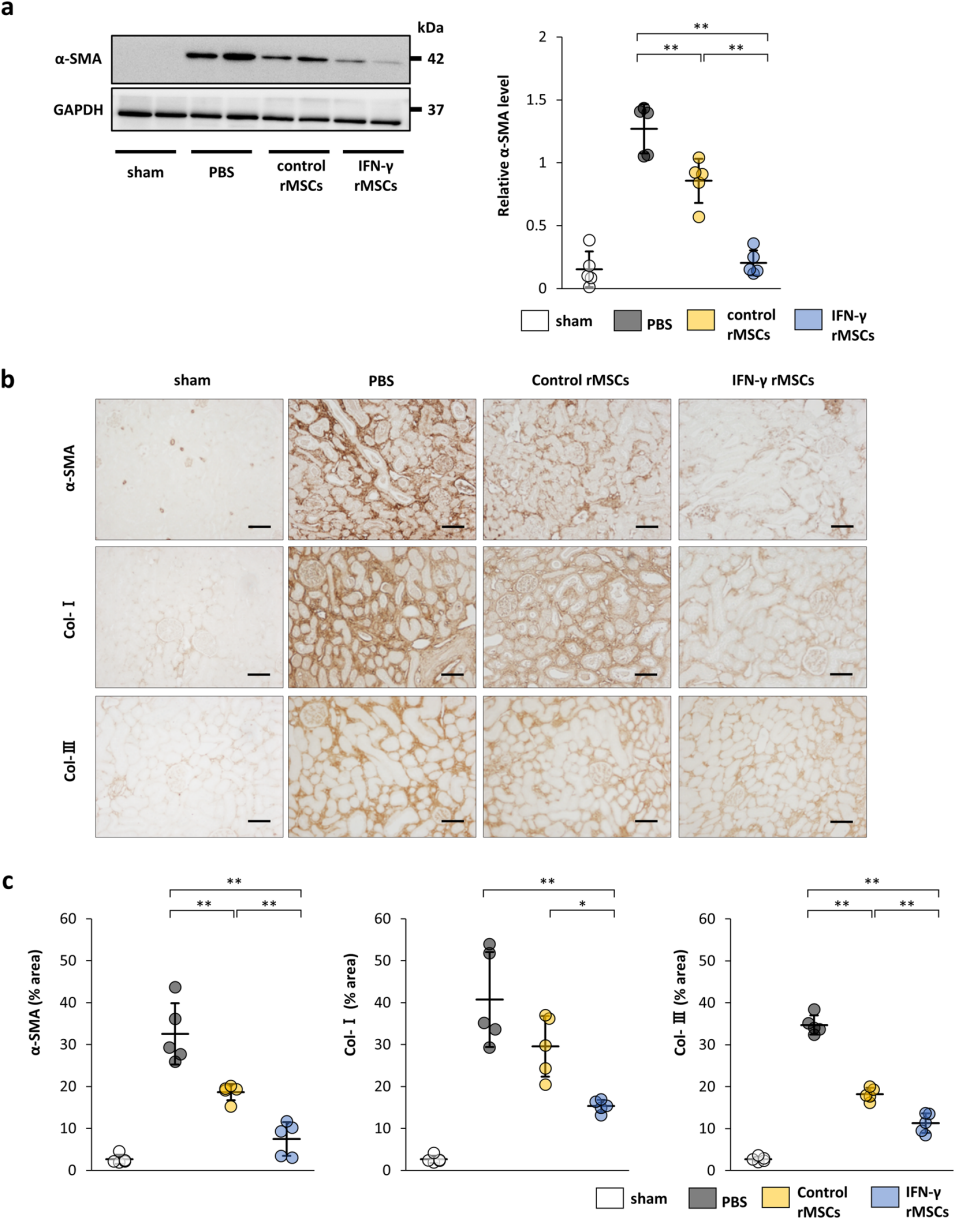
Anti-fibrotic effects of IFN-γ-treated MSCs in unilateral ureter obstruction (UUO) rats. Rat MSCs with or without IFN-γ treatment were injected at 4 days after the UUO procedure. After 11 days, protein levels of fibrosis markers were evaluated by western blot (**a**) and immunohistochemical (**b**,**c**) analyses. (**a**) Western blot analysis of α-SMA in the rat kidney cortex. Protein levels were normalized to GAPDH levels (n = 5 in each group). The blots are the cropped images from different parts of the same gel. Full-length gel images are provided in the supplementary file. The samples derive from the same experiment and that gels were processed in parallel. (**b**) Representative immunohistochemical staining of α-SMA, Col-I, and Col-III in rat kidney sections (scale bar, 100 µm). (**c**) Quantification of α-SMA-, Col-I-, and Col-III-positive areas (n = 5 in each group). Data are presented as the mean ± SD. **p < 0.01. Sham, non-UUO procedure; Other abbreviations are defined in the Fig. 2 legend.

A UUO model was used to confirm the anti-fibrotic effect of IFN-γ rMSCs. Four days after UUO, rats were injected with PBS, control rMSCs, or IFN-γ rMSCs through the tail vein. Seven days after injection, the rats were sacrificed and evaluated for renal fibrosis. Western blotting showed that expression of α-SMA was induced by UUO in the PBS group. The protein levels of α-SMA were suppressed by injection of control rMSCs, and the suppression of α-SMA levels by IFN-γ rMSCs was more significant than that by control rMSCs (Fig. 4a). Immunostaining also revealed increases in α-SMA, Col-I, and Col-III-positive areas in the PBS group (Fig. 4b,c). The α-SMA and Col-III-positive areas were suppressed by injection of control rMSCs. Administration of control rMSCs also reduced Col-I-positive area, but it did not reach significance (*p* = 0.08). Stronger suppressive effects on fibrosis were observed in rats injected with IFN-γ rMSCs compared with control rMSCs (Fig. 4b,c).

### Conditioned medium from IFN-γ-preconditioned MSCs strongly inhibits TGF-β1/Smad signaling in a paracrine fashion

**Figure 5:**
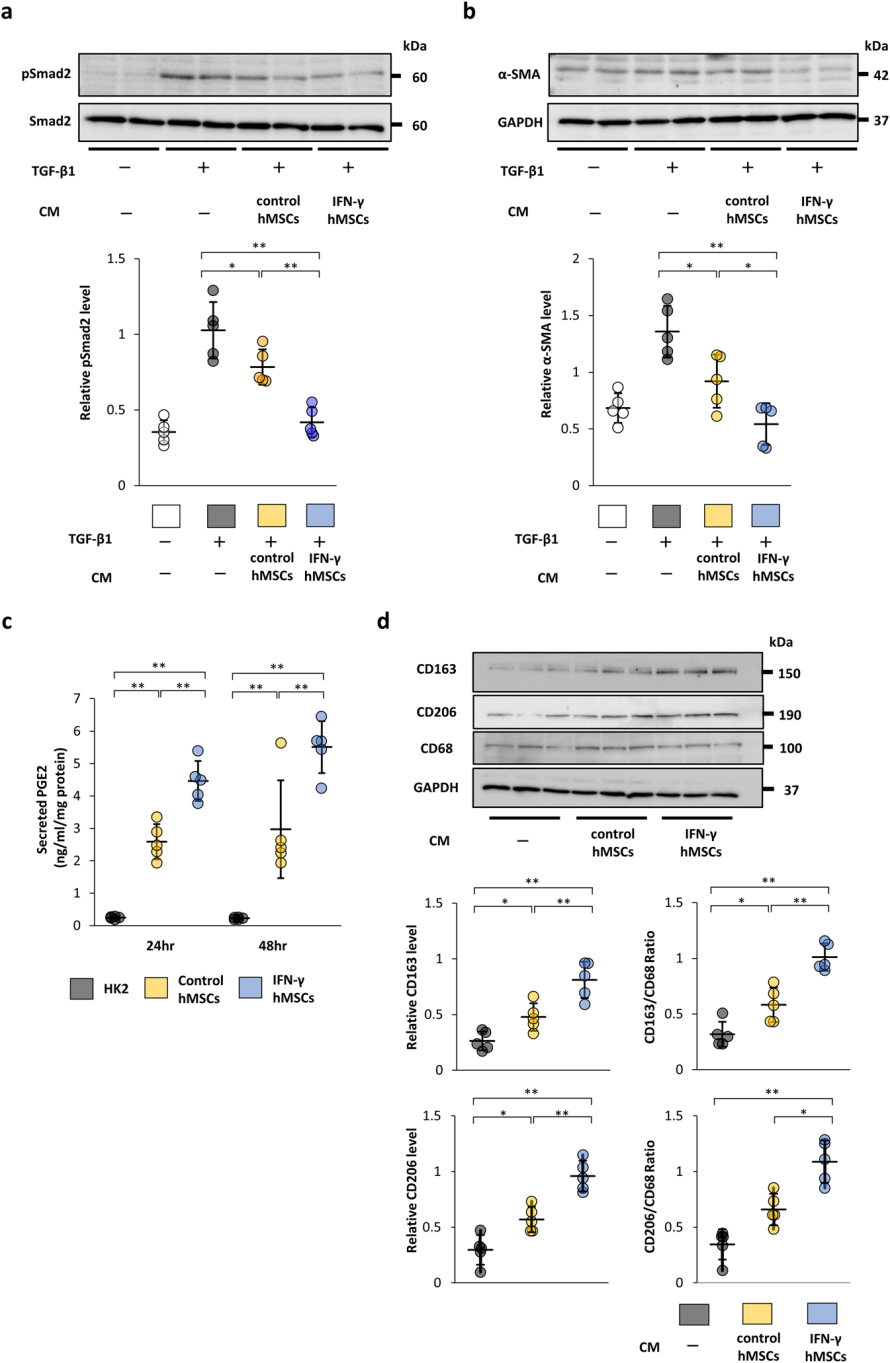
Paracrine effects of IFN-γ-treated human MSCs (hMSCs) on the TGF-β/Smad signaling pathway and phenotypic changes of macrophages. (**a**, **b**) After HK-2 cells, a human proximal tubular cell line*,* were incubated with conditioned medium (CM) obtained from IFN-γ-treated or untreated hMSCs for 24 h, the cells were treated with TGF-β1 for 30 min (**a**) or 24 h (**b**). (**a**) Western blot analysis of phosphorylated Smad2 (p-Smad2) and Smad2 in HK-2 cells. p-Smad2 protein levels were normalized to Smad2 levels (n = 5 in each group). (**b**) Western blot analysis of α-SMA in HK-2 cells. α-SMA protein levels were normalized to GAPDH levels (n = 5 in each group). The blots are the cropped images from different parts of the same gel. Full-length gel images are provided in the supplementary file. The samples derive from the same experiment and that gels were processed in parallel. Control hMSCs, CM from hMSCs without IFN-γ treatment; IFN-γ hMSCs, CM from hMSCs treated with IFN-γ. (**c**) Concentration of PGE2 in CM from hMSCs was evaluated by an ELISA at 24 or 48 h after IFN-γ stimulation. HK-2 cells were used as a negative control (n = 5 in each group). HK-2, CM from HK-2 cells. (**d**) Phenotypic changes of macrophages were evaluated by CD163 and CD68 protein levels. To induce differentiation of monocytic THP-1 cells into macrophages, the cells were treated with PMA for 48 h. The induced THP-1 cells were incubated with CM from IFN-γ-treated or untreated hMSCs. After 48 h, the cells were harvested and subjected to western blot analysis of CD163, CD206 and CD68. CD163 and CD206 protein levels were normalized to the GAPDH and CD68 levels (n = 5 in each group). The blots are the cropped images from different parts of the same gel. Full-length gel images are provided in the supplementary file. The samples derive from the same experiment and that gels were processed in parallel. Data are presented as the mean ± SD. *p < 0.05, **p < 0.01.

The TGF-β1/Smad signaling pathway is known to induce renal fibrosis. To investigate whether MSCs treated with IFN-γ strongly inhibit TGF-β1/Smad signaling in a paracrine fashion, we used conditioned medium (CM) from human bone marrow MSCs (hMSCs) treated with IFN-γ (IFN-γ hMSCs) or untreated hMSCs (control hMSCs). The protein levels of phosphorylated Smad2 (p-Smad2) and α-SMA in HK-2 cells were increased by TGF-β1 stimulation, and the increased levels were reduced by incubation in CM from control hMSCs (Fig. 5a,b). CM from IFN-γ hMSCs showed stronger suppressive effects than that from control hMSCs (Fig. 5a,b). These findings suggest that IFN-γ-preconditioned hMSCs strongly inhibit the TGF-β1/Smad signaling pathway.

### IFN-γ enhances secretion of PGE2 from MSCs

It has been reported that secretion of PGE2 from MSCs exerts anti-inflammatory effects^24^. Thus, the amount of PGE2 contained in CM from IFN-γ hMSCs and control hMSCs was measured by an enzyme-linked immunosorbent assay (ELISA). The amount of PGE2 was significantly elevated in CM from control hMSCs. Furthermore, the levels of secreted PGE2 were more markedly increased in CM from IFN-γ hMSCs (Fig. 5c).

### IFN-γ-preconditioned MSCs promote a change in the phenotype of macrophages from M1 to M2

The phenotypic change of macrophages from M1 (proinflammatory macrophages) to M2 is considered as one of the reasons for the anti-inflammatory effects of MSCs^27,28^. We investigated whether MSCs treated with IFN-γ affect the phenotypic change of macrophages using monocytic THP-1 cells. THP-1 cells were stimulated with phorbol 12-myristate 13-acetate (PMA) to induce differentiation into macrophages. Then, the medium was replaced with 0.1% fetal bovine serum (FBS)-containing medium or CM from either control hMSCs or IFN-γ hMSCs. Forty-eight hours after medium replacement, the cells were collected and expression of CD163, CD206 and CD68 was evaluated. Although there was no significant difference in the protein levels of CD68, those of CD163 and CD206 were upregulated by CM from control hMSCs (Fig. 5d). Furthermore, CM from IFN-γ hMSCs resulted in stronger induction of CD163 and CD206 compared with that from hMSCs, indicating increased infiltration of CD163 and CD206-positive immunosuppressive macrophages.

### Knockdown of prostaglandin E synthase weakens the ability of IFN-γ-preconditioned MSCs to change the phenotype of macrophages to immunosuppressive M2

**Figure 6:**
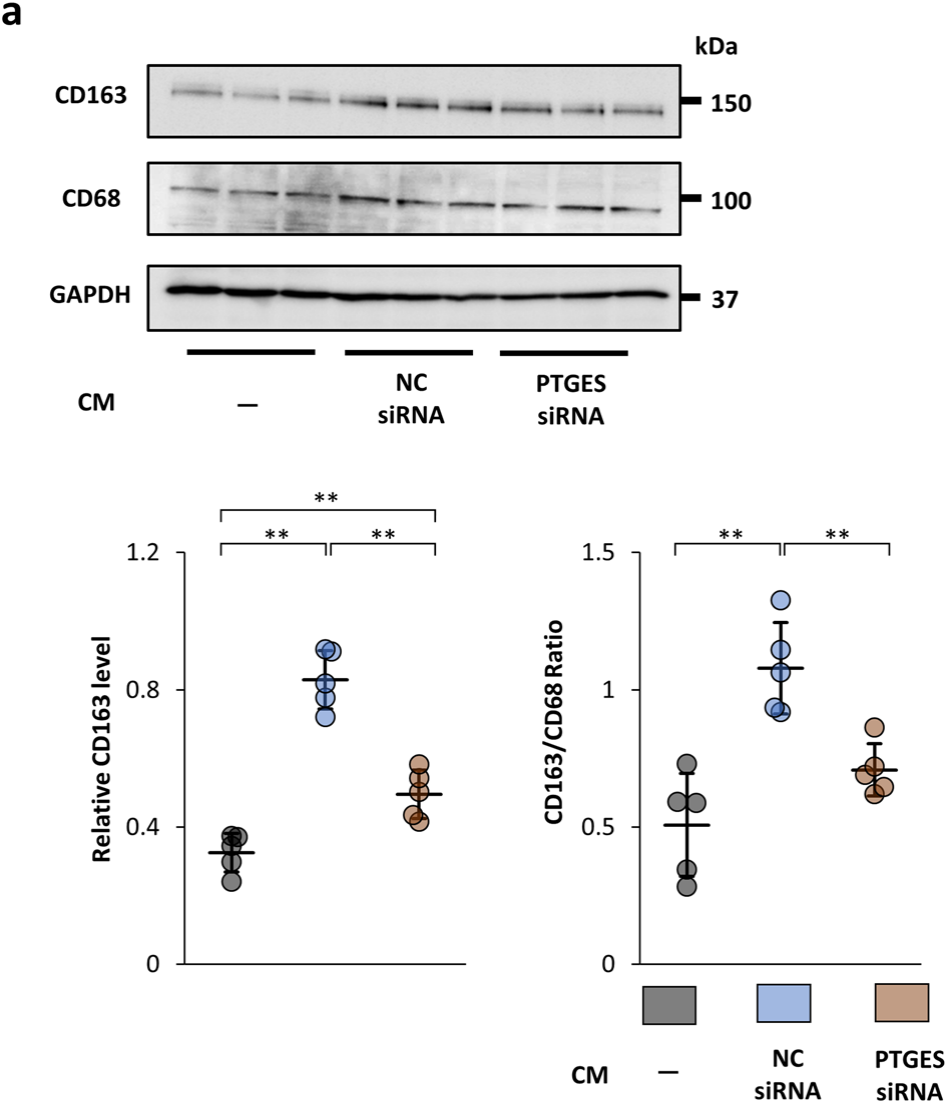
Suppressive effects of prostaglandin E synthase (PTGES) siRNA in IFN-γ-treated MSCs on the phenotypic change of macrophages from M1 to M2. (**a**) THP-1 monocytes were stimulated by PMA for 48 h and then incubated with CM from IFN-γ-treated hMSCs transfected with negative control (NC) siRNA or PTGES siRNA. After 48 h, the cells were collected and subjected to western blot analysis of CD163 and CD68. CD163 protein levels were normalized to GAPDH and CD68 levels (n = 5 in each group). The blots are the cropped images from different parts of the same gel. Full-length gel images are provided in the supplementary file. The samples derive from the same experiment and that gels were processed in parallel. Data are presented as the mean ± SD. *p < 0.05, **p < 0.01.

We hypothesized that the potent anti-fibrotic and anti-inflammatory effects of IFN-γ MSCs were due to increased secretion of PGE2. First, we investigated whether prostaglandin E synthase (PTGES) siRNA affected the anti-fibrotic ability of IFN-γ hMSCs using HK2 cells and CM. hMSCs were transfected with PTGES siRNA or negative control siRNA (NC siRNA) for 12 h. After transfection, hMSCs were stimulated by IFN-γ for 24 h. IFN-γ hMSCs transfected with PTGES siRNA showed significant decreases in PTGES mRNA (Supplementary Fig. S1a). Although the protein levels of α-SMA in HK-2 cells were decreased by incubation in CM from IFN-γ hMSCs transfected with NC siRNA, there was no significant difference between NC siRNA and PTGES siRNA groups (Supplementary Fig. S1b). Next, to identify the effects of PTGES siRNA on the phenotypic change of macrophages, THP-1 monocytes were stimulated with PMA. After 48 h, the medium was replaced with 0.1% FBS-containing medium or CM from IFN-γ hMSCs transfected with NC siRNA or PTGES siRNA. After the medium replacement, the cells were collected to evaluate expression of CD163 and CD68. The protein levels of CD163 were increased by CM from IFN-γ hMSCs transfected with NC siRNA (Fig. 6a). However, the upregulation of CD163 was suppressed by CM from IFN-γ hMSCs transfected with PTGES siRNA.

### Knockdown of PTGES weakens the anti-fibrotic effect of IFN-γ-preconditioned MSCs in IRI models

**Figure 7:**
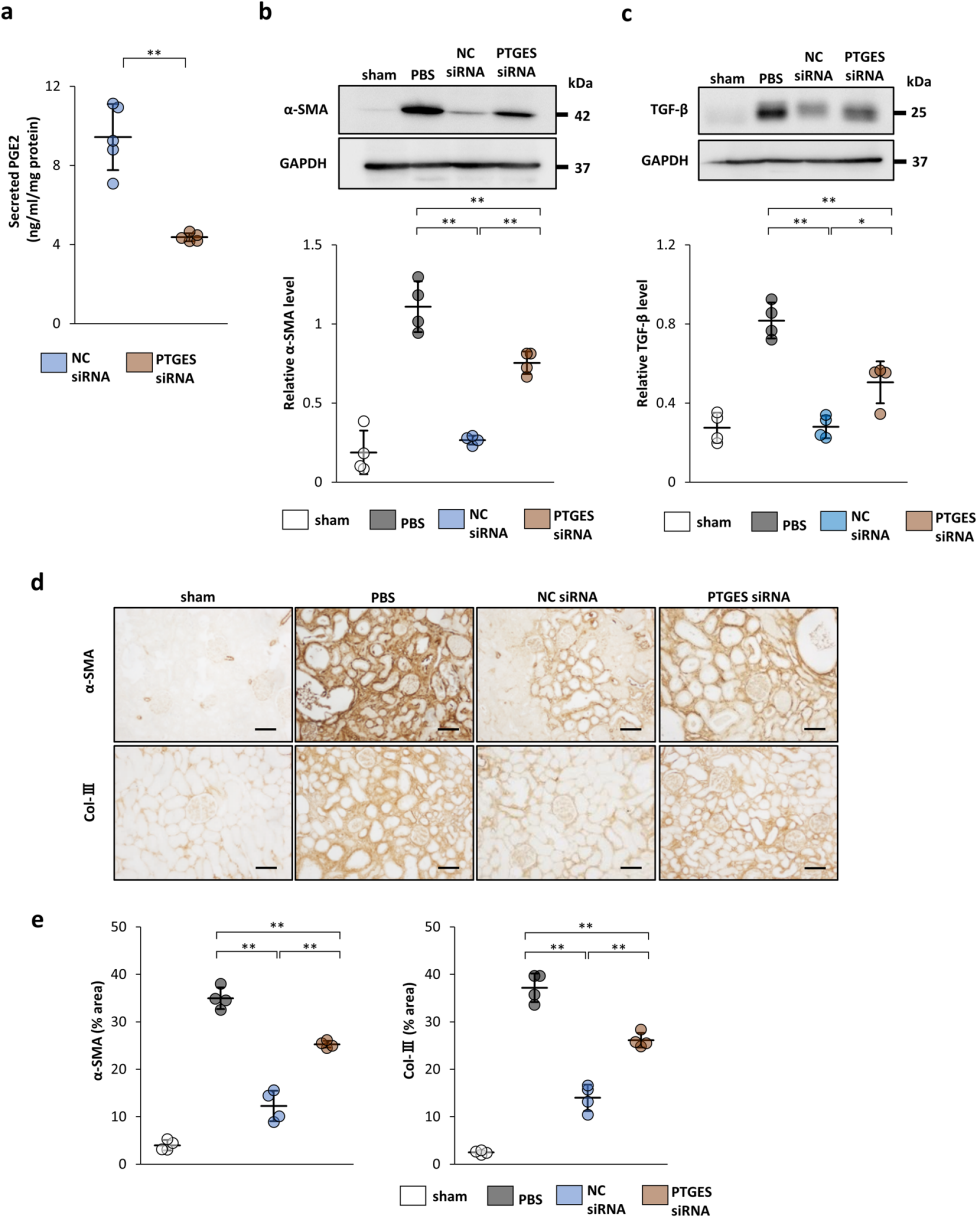
Inhibitory effects of PTGES siRNA on the anti-fibrotic effects of IFN-γ-treated rMSCs in IRI rats. After rMSCs transfected with NC siRNA or PTGES siRNA were treated with IFN-γ, the cells were injected into rats immediately after IRI induction. Twenty-one days later, protein levels of fibrosis markers in the kidney were evaluated by western blot and immunohistochemical analyses. (**a**) Secreted PGE2 levels in CM from IFN-γ-treated rMSCs transfected with NC siRNA or PTGES siRNA were evaluated by an ELISA (n = 5 in each group). (**b**, **c**) Western blot analysis of α-SMA and TGF-β1 in the rat kidney cortex of IRI rats. Protein levels were normalized to GAPDH levels (n = 4 in each group). The blots are the cropped images from different parts of the same gel. Full-length gel images are provided in the supplementary file. The samples derive from the same experiment and that gels were processed in parallel. Sham, non-IRI procedure; PBS, PBS injection; NC siRNA, injection of IFN-γ rMSCs transfected with NC siRNA; PTGES siRNA, injection of IFN-γ rMSCs transfected with PTGES siRNA. (**d**) Representative immunohistochemical staining of α-SMA and Col-III in kidney sections (scale bar, 100 µm). (**e**) Quantification of α-SMA- and Col-III-positive areas (n = 4 in each group). Data are presented as the mean ± SD. *p < 0.05, **p < 0.01.

IFN-γ-precondition enhanced the expression of PTGES mRNA in rMSCs (Supplementary Fig. S2a) and IFN-γ rMSCs transfected with PTGES siRNA showed significant decreases in PTGES mRNA and secreted PGE2 (Supplementary Fig. S2b, Fig. 7a). PTGES siRNA-transfected IFN-γ rMSCs (PTGES siRNA IFN-γ rMSCs) and NC siRNA-transfected IFN-γ rMSCs (NC siRNA IFN-γ rMSCs) were administered to IRI rats to examine whether PTGES siRNA affects the anti-fibrotic effect of IFN-γ rMSCs. After preparation of IRI rats, either type of rMSCs were injected into the abdominal aorta, and 21 days later, rats were sacrificed to evaluate fibrosis. Western blotting revealed that the protein levels of α-SMA and TGF-β1 were decreased by NC siRNA IFN-γ rMSCs. However, these decreases were weakened by PTGES siRNA IFN-γ rMSCs (Fig. 7b,c). Similarly, immunostaining showed strong suppression of α-SMA and Col-III-positive areas by NC siRNA IFN-γ rMSCs. These anti-fibrotic effects were attenuated by PTGES siRNA IFN-γ rMSCs (Fig. 7d,e). These results support the hypothesis that the potent renal anti-fibrotic effect of IFN-γ MSCs depends on increased secretion of PGE2.

## Discussion

**Figure 8:**
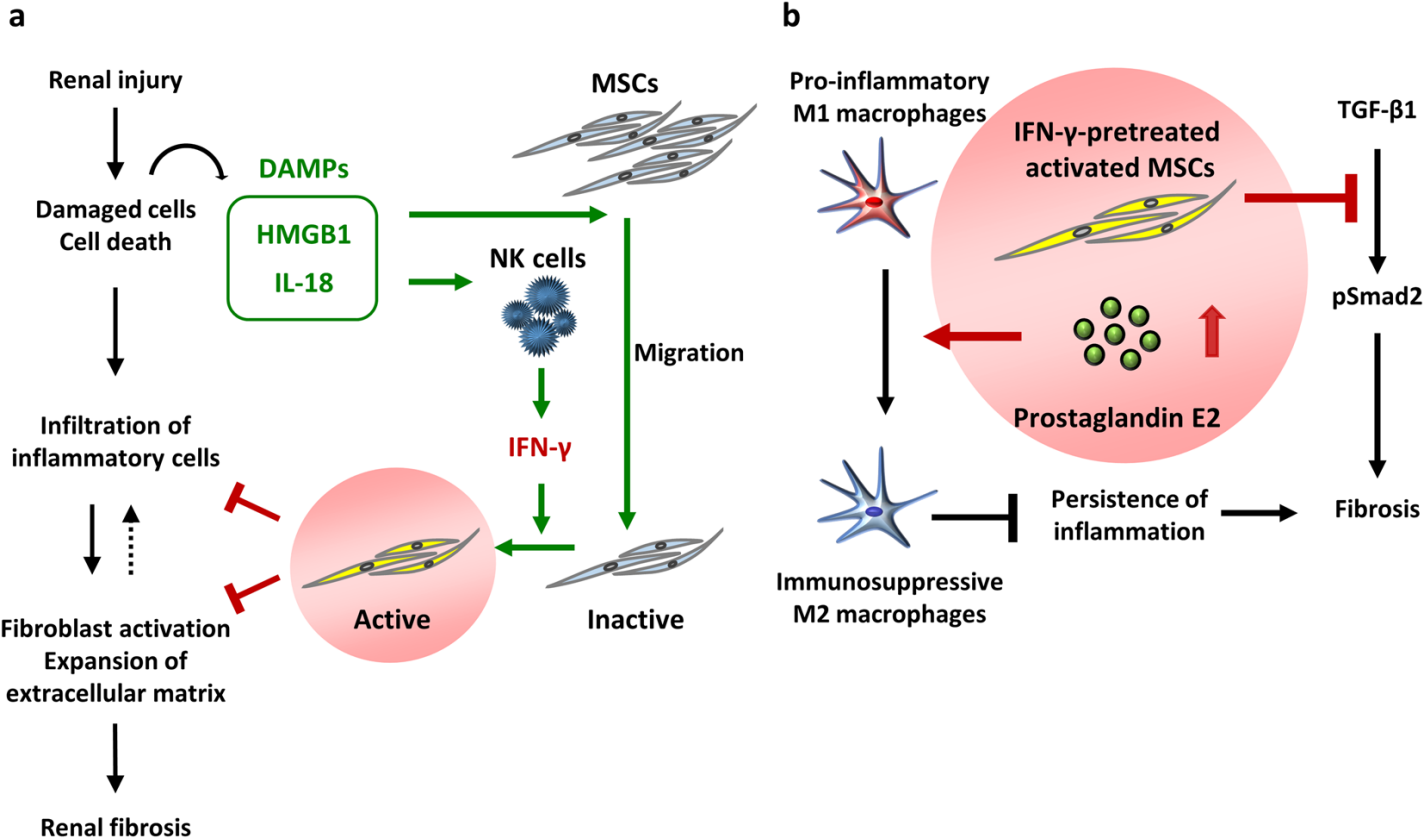
DAMPs and the effects of MSCs treated with IFN-γ on renal fibrosis. (**a**) HMGB1 and IL-18 are members of DAMPs—HMGB1 was reported to promote the migration of MSCs, whereas IL-18 contributed to the secretion of IFN-γ. IFN-γ secreted from natural killer cells in injured tissues activates MSCs that exert anti-inflammatory effects. However, such activation require a long period of time, which delays the effect of MSCs administered for therapeutic purposes. (**b**) IFN-γ stimulation promotes the secretion of prostaglandin E2 from MSCs, and increased prostaglandin E2 induces polarization of immunosuppressive CD163-positive macrophages, suppressing the persistence of inflammation. MSCs treated with IFN-γ also directly inhibit profibrogenic TGF-β1/Smad signaling, leading to the prevention of fibrosis.

Here, we provide the first evidence that administration of MSCs treated with IFN-γ strongly ameliorates renal fibrosis and inflammation in rat IRI and UUO models compared with that of untreated MSCs. Furthermore, CM from MSCs stimulated by IFN-γ inhibits TGF-β/Smad signaling in vitro. IFN-γ stimulation promotes the secretion of PGE2 from MSCs, and increased PGE2 induces polarization of immunosuppressive CD163 and CD206-positive macrophages. Knockdown of PTGES attenuates the anti-fibrotic effect of IFN-γ-treated MSCs in IRI rats. These findings suggest that MSCs treated with IFN-γ have a great potential to suppress renal fibrosis (Fig. 8a).

DAMPs, such as HMGB1 and IL-18, are involved in the induction and repression of inflammation^6–8^. HMGB1 released from damaged tissues cooperates with cytokines, such as those secreted from macrophages, to induce inflammation^33–35^. HMGB1 is also involved in inducing IL-18 production^8^. Induced IL-18 promotes secretion of IFN-γ from natural killer cells^21^. When MSCs cultured under normal conditions are administered to renal injured rats, MSCs can be activated by IFN-γ secreted from natural killer cells in injured tissues and suppress renal inflammation. These processes might require several days, leading to a delay of the onset of MSCs’ immunosuppressive effects. Because anti-inflammation and anti-fibrotic effects of MSCs treated with IFN-γ were enhanced in advance, their strong therapeutic effects appear to be exerted immediately after administration (Fig. 8b).

PGE2 promotes polarization of immunosuppressive M2 macrophages through induction of the cyclic adenosine monophosphate pathway^27^. In this study, MSCs treated with IFN-γ produced increased amounts of PGE2, leading to more pronounced polarization of M2 macrophages. Therefore, we speculated that PGE2 might contribute to the immunosuppressive action of MSCs in vivo. In fact, we found that knockdown of PTGES weakened the anti-fibrotic effect of MSCs treated with IFN-γ in IRI rats. Thus, the upregulation of PGE2 in MSCs plays an important role in anti-inflammation and anti-fibrotic effects of MSCs treated with IFN-γ.

The TGF-β/Smad signaling pathway is considered as one of the processes that cause renal fibrosis. TGF-β is a soluble factor that binds to a cell surface receptor, and phosphorylates and activates Smad2. Phosphorylated Smad2 induces expression of the αSMA gene and promotes fibrosis^36^. The damaged tissues produce TGF-β that induces renal fibrosis via the TGF-β/Smad signaling pathway. Whereas, TGF-β exerts anti-inflammatory effects, and TGF-β blockade might lead to autoimmune disease^37^. Thus, inhibition of the TGF-β/Smad signaling pathway, but not TGF-β itself, might be a key mechanism by which MSCs treated with IFN-γ suppress renal fibrosis. Therefore, in this study, we investigated whether CM from IFN-γ-preconditioned MSCs strongly inhibits the induction of p-Smad2 and α-SMA by TGF-β. Western blotting showed that CM from IFN-γ-preconditioned MSCs significantly decreased TGF-β-induced fibrotic changes compared with that from untreated MSCs. These results suggest that IFN-γ-preconditioned MSCs exert strong anti-fibrotic effects through direct inhibition of the TGF-β/Smad signaling pathway.

We show that renal fibrosis and inflammation are strongly attenuated by injection of IFN-γ-treated MSCs in IRI and UUO rats. The anti-inflammation and anti-fibrotic effects of MSCs treated with IFN-γ are due to direct inhibition of TGF-β/Smad signaling and enhancement of PGE2 secretion, which polarization of immunosuppressive M2 macrophages. As a result, administration of MSCs treated with in IFN-γ might provide a promising therapeutic approach to prevent the progression of renal fibrosis.

## Methods

### Animals

Male Sprague–Dawley (SD) rats were purchased from Charles River Laboratories Japan (Yokohama, Japan). Six-week-old rats were used to collect bone marrow, and 8-week-old rats were used in in vivo experiments. All experiments were performed according to the “Guide for the Care and Use of Laboratory Animals, 8^th^ ed., 2010” (National Institutes of Health, Bethesda, MD) and approved by the Institutional Animal Care and Use Committee of Hiroshima University (Permit number: A15-66 and A17-75).

### Antibodies

As primary antibodies, we used a rabbit monoclonal anti-HMGB1 antibody (ab79823; Abcam, Cambridge, UK), rabbit monoclonal anti-IL-18 antibody (ab223293; Abcam), rabbit monoclonal anti-IFN-γ antibody (ab133566; Abcam), mouse monoclonal anti-α-SMA antibody (A-2547; Sigma-Aldrich; St. Louis, MO), mouse monoclonal anti- TGF-β1 antibody (sc-130348; Santa Cruz Biotechnology, Santa Cruz, CA), mouse monoclonal anti-glyceraldehyde-3-phosphate dehydrogenase (GAPDH) antibody (G8795; Sigma-Aldrich), rabbit monoclonal anti-p-Smad2 antibody (#3108; Cell Signaling Technology, Danvers, MA), mouse monoclonal anti-Smad2 antibody (#3103; Cell Signaling Technology), rabbit polyclonal anti-Col-I antibody (ab6308; Abcam), rabbit polyclonal anti-Col-III antibody (ab7778; Abcam), rabbit polyclonal anti-CD3 antibody (IR503; Dako, Santa Clara, CA), rabbit polyclonal anti-CD68 antibody (ab125212; Abcam), rabbit polyclonal anti-CD206 antibody (ab64693; Abcam) and rabbit monoclonal anti CD163 antibody (ab182422; Abcam).

### Other materials

Rat and human IFN-γ were obtained from PEPROTECH (Rocky Hill, NJ). A PGE2 high sensitivity ELISA kit was purchased from Enzo Life Science (ADI-930–001; Villeurbanne, France). Dulbecco’s modified Eagle’s medium (DMEM) was obtained from Sigma-Aldrich.

### Cell culture

rMSCs were isolated from bone marrow of the SD rat femur and tibia. rMSCs were cultured in DMEM containing 10% FBS (Sigma-Aldrich) for the primary culture. MSCs were passaged four times before used for administration or in vitro experiments. These cells were confirmed as MSCs by promoting their differentiation into osteocytes and adipocytes with specific differentiation media^38^. Furthermore, we confirmed that standard MSC surface markers CD44 and CD90 were positive in these cells by flow cytometry (Supplementary Fig. S3). hMSCs derived from human bone marrow and human THP-1 monocytes were purchased from Riken BRC (Ibaraki, Japan). HK-2 cells, a human proximal tubular cell line, were obtained from the American Type Culture Collection (Manassas, VA). These cells were cultured as described previously^29^.

### Cell preparation for in vivo experiments

rMSCs (3 × 10^5^ cells/100-mm dish) were seeded and cultured in DMEM supplemented with 10% FBS. At 80% confluence, the medium was replaced by fresh medium with or without 200 ng/ml IFN-γ, and the cells were cultured for 24 h. The cells were resuspended in PBS and subjected to in vivo analyses as IFN-γ rMSCs or control rMSCs.

### Preparation of CM

After hMSCs or rMSCs were grown to 80% confluence in DMEM supplemented with 10% FBS, the medium was replaced by fresh medium with or without 200 ng/ml IFN-γ (Supplementary Fig. S4). Twenty-four hours later, the medium was replaced by DMEM supplemented with 0.1% FBS, which was collected after 24 or 48 h.

### Cell treatment with TGF-β1

After incubation in DMEM supplemented with 0.1% FBS, CM from control hMSCs, or CM from hMSCs treated with IFN-γ for 24 h, human HK-2 cells were treated with 10 ng/ml recombinant human TGF-β1 (R&D Systems, Minneapolis, MN). Thirty minutes or 24 h later, the cells were collected and subjected to analysis of TGF-β1-induced fibrotic changes.

### Polarization of M2 macrophages

To induce differentiation of THP-1 monocytes into M1 macrophages, THP-1 cells were stimulated by 160 nM phorbol 12-myristate 13-acetate (Sigma-Aldrich) for 48 h. The medium was replaced by DMEM supplemented with 0.1% FBS, control hMSC-CM, or IFN-γ hMSC-CM. After 48 h, the cells were collected and subjected to western blot analysis of CD163 and CD68.

### Transfection with PTGES siRNA

rMSCs and hMSCs were transfected with 20 nM siRNA against PTGES (s133104 and s18305; Applied Biosystems, Waltham, MA) or negative control siRNA (4,390,843; Applied Biosystems) using Lipofectamine 2000 Transfection Reagent (Thermo Fisher Scientific). Cells were cultured in DMEM supplemented with 10% FBS for 5 days and then subjected to in vitro and in vivo analyses.

### Preparation of an IRI model and MSC administration

A rat IRI model, an acute kidney injury (AKI) model, was used to analyze the progression from AKI to CKD. Rats were anesthetized by intraperitoneal injection of three types of mixed anesthetic agents (butorphanol, medetomidine, and midazolam). The left renal artery was clamped to induce ischemia. Sixty minutes later, the clamp was opened and reperfusion of blood was confirmed. After confirmation of reperfusion, PBS (vehicle), control MSCs, or IFN-γ rMSCs (5 × 10^5^ cells/rat) were injected into the abdominal aorta. After 7 or 21 days, the rats were sacrificed and their kidneys were collected to evaluate inflammation and chronic fibrosis.

### Preparation of a UUO model and MSC administration

A UUO model, which is an experimental renal fibrosis model, was prepared to investigate the anti-fibrotic effect of MSCs. Rats were anesthetized by the same procedure described for the IRI model. The left ureter was exposed after an abdominal midline incision and ligated to induce UUO. At 4 days after the UUO operation, PBS, control rMSCs, or IFN-γ rMSCs (2.5 × 10^6^ cells/rat) were injected into the tail vein. After 7 days, the rats were sacrificed and examined for renal fibrosis.

### Western blotting

Sample preparation and western blotting were performed as described previously^29^. The protein bands were quantified using ImageJ software (version 1.47v; National Institutes of Health) and normalized to GAPDH levels.

### Immunohistochemical analysis

Left kidneys of IRI and UUO rats were fixed in 10% formaldehyde for 18 h. Fixed samples were embedded in paraffin and cut into 4 µm-thick sections. Immunohistochemical staining of kidneys was performed for light microscopic observation. Five fields (× 100) of the renal cortex were selected randomly. CD3-, CD68-, and CD163-positive cells and areas positive for α-SMA, Col-I, and Col-III were assessed using ImageJ software.

### ELISA

To evaluate the amount of PGE2 in CM, the ELISA was performed according to the manufacturer’s instructions. PGE2 concentrations were normalized to the total protein content of MSCs.

### Quantitative real-time reverse transcription-PCR

RNA extraction and quantitative reverse transcription-PCR were performed as described previously^39^. mRNA levels were normalized to the level of 18 s rRNA. Primers and TaqMan probes (TaqMan Gene Expression Assay) were obtained from Applied Biosystems (Foster City, CA). The probe set ID for rat PTGES is Rn00572047_m1 and for human PTGES is Hs00610420_m1.

### Flow cytometric analysis

To confirm MSC surface markers, flow cytometric analysis was performed as previously described^29^. The following antibodies were used: anti-rat CD44 IgG antibody (MCA643FA; BioRad, Hercules, CA), anti-rat CD90 IgG antibody (561,973; Becton, Dickinson and Company, Franklin Lakes, NJ), and anti-rat CD45 IgG antibody (202,205; BioLegend, San Diego, CA). The stained MSCs were analyzed using a BD FACSVerse (Becton, Dickinson and Company). Data were assessed by FlowJo software (FlowJo, LLC; Ashland, OR).

### Statistical analysis

All results are expressed as the mean ± standard deviation (SD). Statistical analysis for multiple comparisons was performed using one-way ANOVA followed by Bonferroni’s post-hoc test. The Student’s t-test was performed to compare the difference between two groups. *p* < 0.05 was defined as statistically significant.

## Supplementary information

Supplementary Information.

## Data Availability

The data that support the findings of this study are available from the corresponding author upon reasonable request.
