# Supplementary material for: Interferon-γ enhances the therapeutic effect of mesenchymal stem cells on experimental renal fibrosis
Source: Sci Rep. 2021 Jan 13;11:850. doi: 10.1038/s41598-020-79664-6 (PMC7807061; doi:10.1038/s41598-020-79664-6)

Supplementary material

Interferon-γ enhances the therapeutic effect of mesenchymal stem cells on experimental renal fibrosis

Ryo Kanai[1], Ayumu Nakashima[1,2], Shigehiro Doi[1], Tomoe Kimura[1], Ken Yoshida[1], Satoshi Maeda[2,3], Naoki Ishiuchi[1], Yumi Yamada[1], Takeshi Ike[1], Toshiki Doi[1], Yukio Kato[2,3] & Takao Masaki[1]

[1]Department of Nephrology, Hiroshima University Hospital, 1-2-3 Kasumi, Minami-ku, Hiroshima, Hiroshima 734-8551, Japan

[2]Department of Stem Cell Biology and Medicine, Graduate School of Biomedical & Health Sciences, Hiroshima University, 1-2-3 Kasumi, Minami-ku, Hiroshima, Hiroshima 734-8553, Japan

[3]TWOCELLS Company, Limited, 16-35 Hijiyama-honmachi, Minami-ku, Hiroshima, 732-0816, Japan

This file includes supplementary figure S1, S2, S3 and S4 and western blotting uncropped gel images.

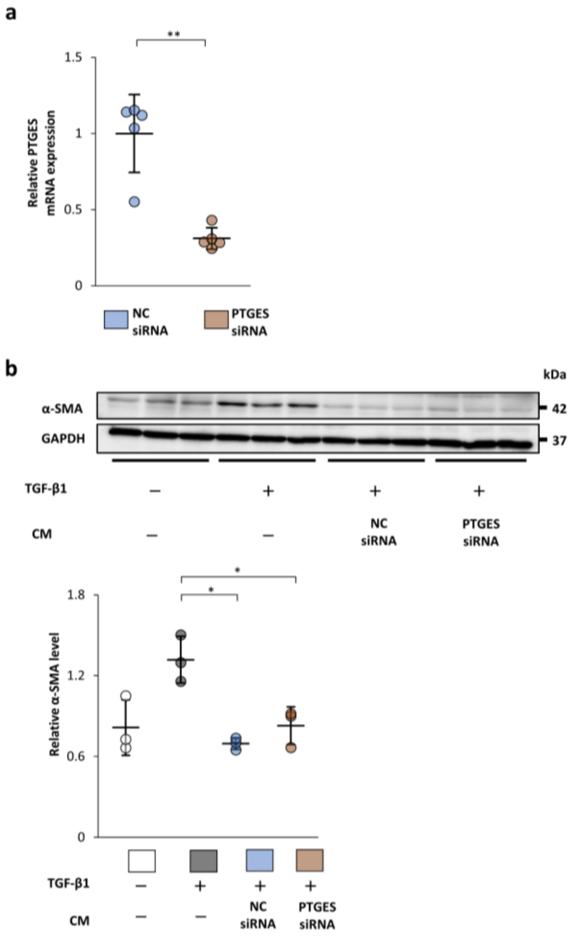

Supplementary figure S1. Effects of PTGES siRNA on the anti-fibrotic effects of IFN-γ-treated hMSCs *in vitro*. (**a**) After hMSCs were transfected with NC siRNA or PTGES siRNA, the cells were treated with IFN-γ, and then PTGES mRNA levels were evaluated by real-time PCR. PTGES mRNA levels were normalized to the level of 18S rRNA (n = 5 in each group). (**b**) After HK-2 cells were incubated with conditioned medium (CM) from IFN-γ-treated hMSCs transfected with NC siRNA or PTGES siRNA for 24 hours, the cells were treated with TGF-β1. The figure and graph show western blot analysis of α-SMA in HK-2 cells. α-SMA protein levels were normalized to GAPDH levels (n = 3 in each group). Data are presented as the mean ± SD. *$p < 0.05$, **$p < 0.01$.

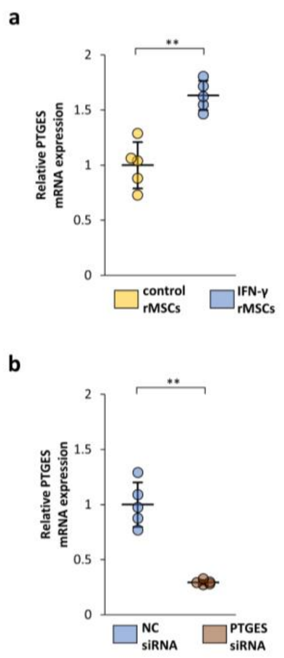

Supplementary figure S2. Expression of PTGES mRNA in rMSCs. (**a**) PTGES mRNA levels in rMSCs treated with or without IFN-γ were evaluated by real-time PCR. PTGES mRNA levels were normalized to 18S rRNA levels (n = 5 in each group). (**b**) After rMSCs were transfected with NC or PTGES siRNAs, rMSCs were treated with IFN-γ, and then PTGES mRNA levels were evaluated by real-time PCR. PTGES mRNA levels were normalized to the level of 18S rRNA (n = 5 in each group). Data are presented as the mean ± SD. $^{**}p < 0.01$.

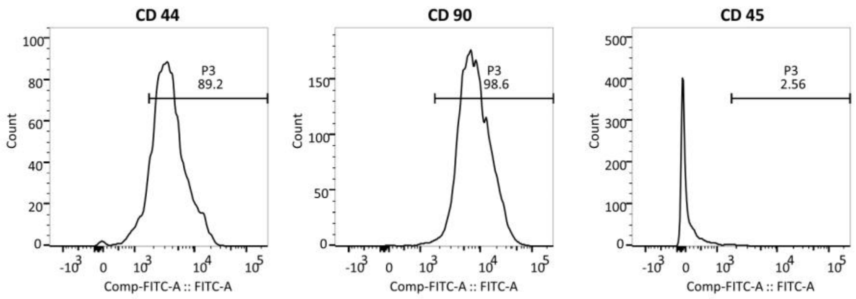

Supplementary figure S3. Cell surface markers on rMSCs. Flow cytometry showing the expression of surface markers on bone marrow-derived rMSCs.

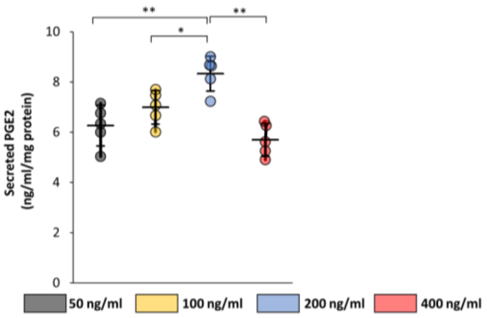

Supplementary figure S4. The amount of PGE2 from hMSCs over different concentrations of IFN-γ. The amount of PGE2 in conditioned medium (CM) from hMSCs with 50, 100, 200, and 400 ng/ml IFN-γ were analyzed by ELISA (n = 5 in each group). PGE2 secretion was significantly elevated in CM from 200 ng/ml IFN-γ-treated human MSCs. Data are presented as the mean ± SD. *p < 0.05, **p < 0.01.

Western blotting uncropped gel images

Figure 1

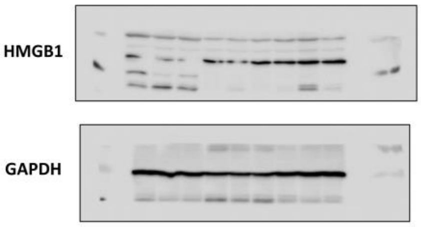

Figure 2

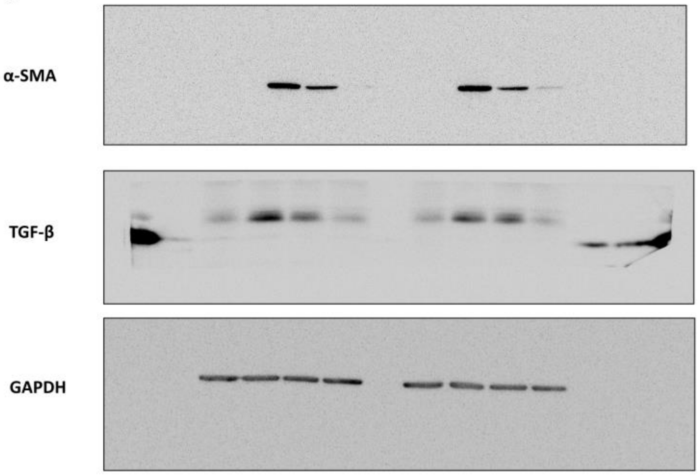

Figure 4

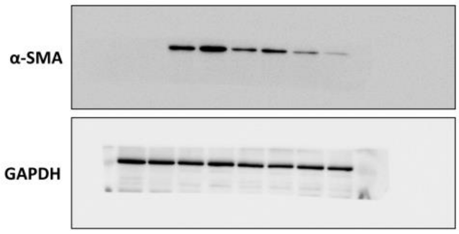

Figure 5

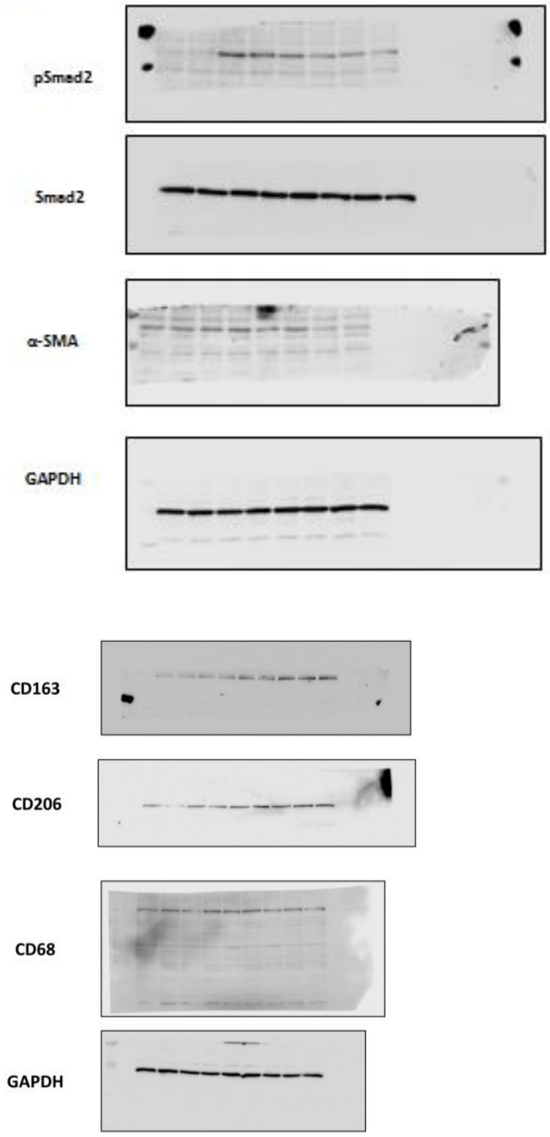

Figure 6

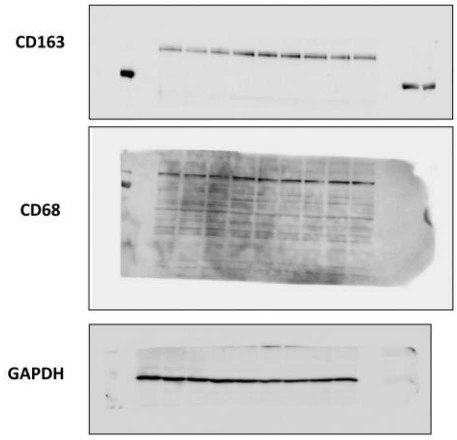

Figure 7

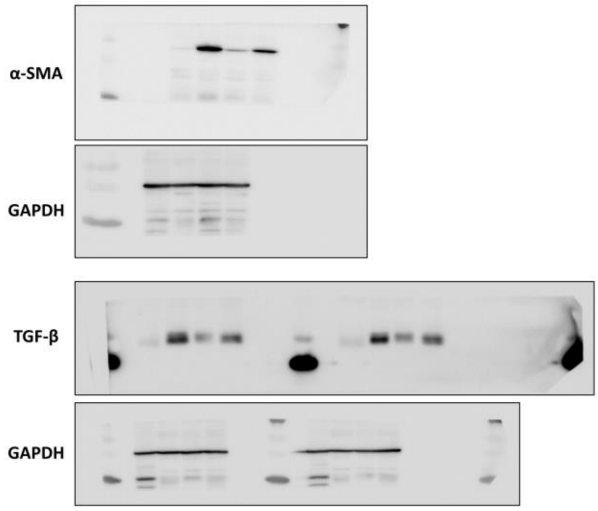

Supplementary Figure S1

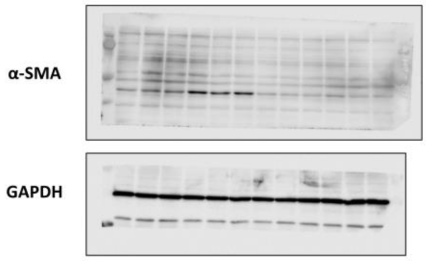

Supplement: Supplementary file 1 — Supplementary Information. [file 41598_2020_79664_MOESM1_ESM.pdf]
